# Advances in Multitarget Therapeutic Approaches for Immune-Mediated Glomerular Diseases

**DOI:** 10.3390/life15020243

**Published:** 2025-02-06

**Authors:** Luminita Voroneanu, Andreea Covic, Vladimir Tesar, Mehmet Kanbay, Adrian Covic

**Affiliations:** 1 Faculty of Medicine, University of Medicine and Pharmacy “Grigore T Popa”, 700115 Iasi, Romania; andreea.covic@gmail.com (A.C.); accovic@gmail.com (A.C.); 2 Nephrology Clinic, Dialysis, and Renal Transplant Center, “C.I. Parhon” University Hospital, 700503 Iasi, Romania; 3 General University Hospital, Charles University, 700820 Prague, Czech Republic; vladimir.tesar@vfn.cz; 4 Division of Nephrology, Department of Internal Medicine, Koc University School of Medicine, 34010 Istanbul, Turkey; mkanbay@yahoo.com; 5 Academy of Romanian Scientists (AOSR), 3 Ilfov Street, Sector 5, 50044 Bucharest, Romania

**Keywords:** glomerulonephritis, multitarget therapy, rituximab, cyclophosphamide

## Abstract

Glomerulonephritis (GN) encompasses a diverse group of immune-mediated diseases that damage the glomerular component of the nephron. While kidney biopsy remains the gold standard for diagnosis, it often fails to provide adequate insight into the underlying etiology of GN. Current classification systems have limited our understanding of the disease’s pathophysiology and hinder the development of targeted therapies. Immunosuppressive treatments, such as glucocorticoids, calcineurin inhibitors, cyclophosphamide, and rituximab, remain the mainstay of therapy, though many patients fail to achieve remission or experience significant adverse effects. Moreover, the complex and multifactorial nature of GN pathogenesis calls for more refined therapeutic approaches. In recent years, multitarget therapies—combining different immunosuppressive agents targeting distinct immune pathways—have emerged as promising alternatives. Evidence suggests that multitarget therapy may offer superior outcomes compared to standard treatments. Despite early success, further studies are needed to optimize these regimens, reduce toxicity, and extend benefits to a broader range of GN patients. The development of personalized, biomarker-driven treatments, potentially leveraging innovative drug delivery systems and targeted biologics, holds promise for transforming GN care in the future.

## 1. Introduction

Glomerulonephritis (GN) refers to a group of immune-mediated disorders characterized by injury to the glomerular component of the nephron. Kidney biopsy—the gold standard for diagnosis, which provides information regarding immunological activity, chronicity and severity of the disease—offers little information on the etiology of the disease, with different causes often leading to the same lesion pattern. This classification has limited the understanding of immunopathological injury mechanisms and does not provide sufficient information for targeted treatment. Numerous other classifications of glomerular nephropathies have been proposed, of particular interest being the classification based on the cell which is the primary target: (1) podocytopathies—minimal change disease (MCD), focal segmental glomerulosclerosis (FSGS), membranous nephropathy (MN), (2) mesangioproliferative (Immunoglobulin A nephropathy—IgAN) and membranoproliferative GN (typically, but not only lupus nephritis) and (3) necrotizing/crescentic GN (typically Antineutrophil cytoplasmic antibody (ANCA), anti-glomerular basement membrane (anti-GBM).

In most cases, GN cases are associated with an increased risk of severe complications—thromboembolic, infectious or metabolic. The major goal of treatment is to induce complete remission using efficient drugs that cause the fewest adverse reactions. Pathogenic non-immunosuppressive therapies are often insufficient, requiring immunosuppressive treatments to achieve disease remission.

**Figure 1:**
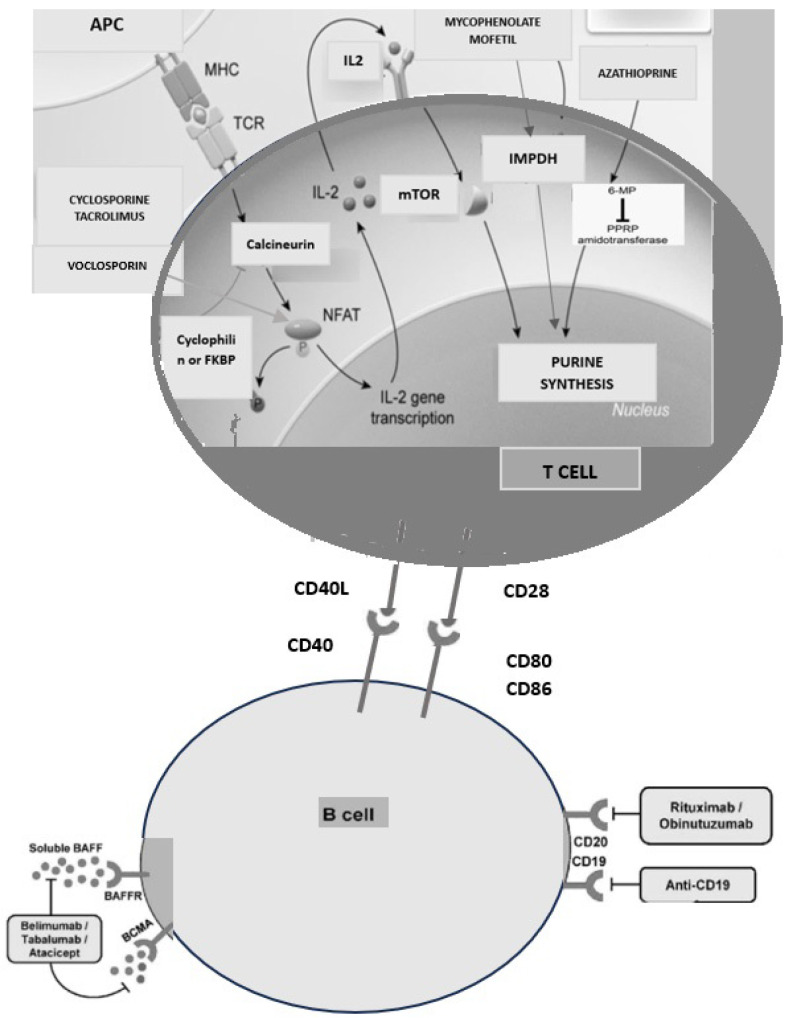
The main component of multitarget therapy and their mechanisms of action.

Currently, approved treatment options for GN include glucocorticoids, calcineurin inhibitors, azathioprine/mycophenolate mofetil (MMF), cyclophosphamide, and B-cell-targeting therapies. Their mechanisms of action are illustrated in Figure 1. The limited number of treatment options reflects both a limited understanding of the disease pathobiology and the rarity of GN. Despite available regimens, many patients fail to achieve clinical or immunological remission and remain at heightened risk for disease progression and complications. For example, up to 60% of patients with membranous nephropathy fail to achieve remission within 12 months; in IgA nephropathy, only 20–50% respond to corticosteroids or immunosuppressive therapy; lupus nephritis (LN) achieves partial or complete remission in 60% of cases following induction therapy and ANCA-associated vasculitis obtain remission rates of 80–90% during induction, though relapse rates reach ~50% within five years, necessitating maintenance therapy [1,2,3,4,5].

Moreover, immunosuppressive therapies are associated with a range of potential side effects that can complicate patient care. The adverse effects of corticosteroid therapy are well described and are both dose- and time-dependent. Cyclophosphamide carries significant risks (bone marrow suppression, increasing the risk of infection and bleeding, hemorrhagic cystitis, bladder cancer or infertility). Rituximab has been associated with infusion-related reactions such as fever, chills, and hypotension during the initial doses. Moreover, it raises concerns due to its non-selective action. The drug not only targets pathogenic B cells involved in the autoimmune process but also eliminates healthy B cells, which can compromise immune function and lead to increased susceptibility to infections [6]. Tacrolimus can lead to nephrotoxicity, while azathioprine may cause bone marrow suppression and liver toxicity. Mycophenolate mofetil is associated with gastrointestinal symptoms, such as nausea and vomiting, and can also affect fetal development, making it contraindicated in pregnancy.

In the past two decades, an unprecedented number of podocyte-directed small molecules were/are investigated in clinical trials, such as agents that target *apolipoprotein L1* (APOL1), transient receptor potential channels (TRPC), complement and reactive oxygen species (ROS) [7]. Innovative nanoparticle-based drug delivery systems are likely to transform the treatment of podocyte-related disorders by improving drug delivery precisely to podocytes, permitting better treatment efficiency [8]. Exosomes, nanoscale extracellular vesicles formed through the invagination of the endosomal membrane, are secreted by most cell types. Kidney-derived exosomes may contribute to glomerular disease pathogenesis or serve as therapeutic resources, especially in MN or LN [9]. This approach to glomerular nephropathy is certainly innovative and adapted to the future, but it is also extremely purist and optimistic. Indeed, we need to adapt our strategy to the future, and based on biomarkers and histological findings, we need individualized therapy. However, despite rigorous and extensive research efforts, truly personalized glomerulopathies-targeted therapies are not yet available.

Until then, our patients continue to face critical complications such as deteriorating estimated glomerular filtration rate (eGFR) and severe, complicated nephrotic syndrome. These challenges underscore the urgency for continued research and innovation in GN treatment. In this context, emerging strategies for remission induction are essential to halt disease progression, prevent complications, and improve long-term outcomes, all while minimizing significant side effects. GN pathogenesis involves a complex interplay of mechanisms, including B cell dysfunction, inflammation, cytokine release, immune signaling pathway dysregulation, and sometimes complement activation. These diverse mechanisms can be targeted with different immunosuppressive agents (we can target inflammation and communication issues with high-dose steroids and anti-tumor necrosis factor—anti-TNF drugs/antibodies; we can target B cells with anti-CD20 and plasma cells with anti-CD38 antibodies and proteosome inhibitors; we can target complement with C5, C3 inhibitors; we can target signaling with various (anti-B-cell activating factor (BAFF) and anti A proliferation-inducing ligand (APRIL), anti-interleukin 2 (IL)2), and with calcineurin inhibitors; in addition, we can target the cell cycle of T/B cells [7]). The search for optimal therapy that balances efficacy with minimal side effects remains critical in treating autoimmune and glomerular diseases.

Given that GN involves multiple underlying mechanisms, a multitarget approach may offer the best strategy, particularly for initial treatment or in resistant or relapsing disease cases. Combining well-established, low-dose agents with a recognized safety profile, each targeting key immunological factors may provide a valuable treatment option. Reducing toxicity by adjusting drug doses can significantly improve patient quality of life. This approach is based on the success seen in kidney transplantation, where a combination of calcineurin inhibitors (such as cyclosporine or tacrolimus), antimetabolites (like MMF), and glucocorticoids has transformed transplant outcomes. These drugs work synergistically to prevent rejection and improve long-term graft survival. For example, the five-year survival rate of kidneys from deceased donors increased from 66.2% in 1996–1999 to 76.3% for those transplanted in 2016 in the U.S. Similarly, survival rates for kidneys from living donors improved from 79.5% to 87.2% for those transplanted in 2016 in the US [10,11].

The purpose of this review is to search for the potential of multitarget therapies in attaining remission, diminishing side effects, and improving long-term outcomes for patients with glomerular diseases.

## 2. What Would Be the Evidence Regarding the Effectiveness of Multitarget Therapies?

LN is a severe complication of systemic lupus erythematosus and a leading cause of mortality among these patients. Approximately 60% of individuals with lupus develop LN and 5–20% of those with LN progress to kidney failure within 10 years [12]. Current standard induction therapies primarily involve the use of MMF or cyclophosphamide combined with glucocorticoids, but these regimens often have suboptimal efficacy in achieving complete renal remission [12]. To address the limitations of these standard therapies, multitarget therapy has emerged as a promising alternative.

The first study on multitarget therapy, published in 2015, involved a randomized open-label trial across 26 renal centers in China. Patients were assigned to either tacrolimus (4 mg/day) plus MMF (1.0 g/day) or intravenous cyclophosphamide (starting at 0.75 g/m^2^ of body surface area every 4 weeks for 6 months). Both groups also received a 3-day pulse of methylprednisolone followed by a tapering course of oral prednisone. After 24 weeks, significantly more patients in the multitarget therapy group achieved complete or partial remission compared to those receiving conventional therapy (83.5% vs. 63.0%, *p* < 0.001) [13]. The median time to overall response was also shorter in the multitarget group (8.9 weeks vs. 13.0 weeks), and the incidence of adverse events was similar between the groups [13].

In an extension of this study, multitarget therapy as a maintenance treatment for LN showed comparable cumulative renal relapse rates to conventional therapies (5.47% vs. 7.62%, respectively; *p* = 0.74). Additionally, renal function remained stable in both groups. Patients who had received multitarget induction therapy continued with the same regimen (tacrolimus 2–3 mg/day, mycophenolate mofetil 0.50–0.75 g/day, prednisone 10 mg/day), while those previously treated with intravenous cyclophosphamide were switched to azathioprine (2 mg/kg/day) plus prednisone (10 mg/day). The multitarget group had fewer adverse events (16.4% vs. 44% for azathioprine; *p* < 0.01) and a lower withdrawal rate due to adverse events (1.7% vs. 8.9% for azathioprine; *p* = 0.02) [14].

Two recent network meta-analyses have compared the efficacy and safety of various immunosuppressive regimens in adults with LN. The first, published in 2023, included 62 trials with 6936 patients [15]. It found that the combination of tacrolimus, mycophenolate, and glucocorticoids achieved the highest rate of total remission, while the combination of voclosporin, mycophenolate, and glucocorticoids showed the best results for complete remission. The second meta-analysis, which included 10 RCTs with 1989 patients, demonstrated that multitarget therapy, obinutuzumab, belimumab, and voclosporin were all superior to standard therapy in achieving complete renal remission [16].

However, since the studies on multitarget therapy were primarily conducted among Chinese patients, further research is needed to determine whether these positive outcomes can be replicated in non-Asian populations.

A new LN guideline published in November 2024 recommends triple therapy as the most preferred treatment for LN [17]. The guideline favors MMF regimens over cyclophosphamide and proposes pulse intravenous methylprednisolone followed by oral glucocorticoids (≤0.5 mg/kg/day, maximum 40 mg/day) with a taper to ≤5 mg/day by 6 months. This regimen should be combined with either mycophenolate plus belimumab, mycophenolate plus calcineurin inhibitors, or low-dose cyclophosphamide plus belimumab. Voclosporin is recommended as a viable option in combination with mycophenolate mofetil and low-dose glucocorticoids for active LN. Voclosporin, a novel calcineurin inhibitor with a dual mechanism of action, suppresses T-cell activation and cytokine production while stabilizing podocytes in the kidney. This recommendation is based on the AURORA Clinical Program, which showed that 40.8% of patients receiving triple therapy achieved complete renal response at 52 weeks compared to 22.5% in the control group (MMF + low-dose glucocorticoids) [18]. Furthermore, over 80% of patients reduced their steroid dose to <2.5 mg/day by 16 weeks.

## 3. More Evidence Has Emerged from MN

Monotherapy with rituximab or calcineurin inhibitors has partial effectiveness, attaining clinical remission in only 60% of patients [1]. In contrast, combination therapy with cyclophosphamide and steroids can achieve better remission rates but can cause short- and long-term side effects associated with prolonged therapy [19]. Treatment failures are more dominant in high-risk patients, with elevated proteinuria levels or/and amplified serum M-type phospholipase A2 receptor (anti-PLA2R) levels. Some studies have demonstrated that multitarget therapy (a combination of rituximab, cyclophosphamide, and corticosteroids) can induce rapid remission, halt disease progression, prevent complications, and improve long-term prognosis.

The first retrospective study included 60 patients with MN (biopsy-proven MN or with positive serum anti-PLA_2_R antibody—74%) with nephrotic syndrome (proteinuria 8.4 g/day) and a median eGFR of 64 mL/min/1.73 m^2^). 30% had a baseline eGFR of 30–60 mL/min/1.73 m^2^, and 13% had a baseline eGFR < 30 mL/min/1.73 m^2^. A large percentage of patients had received prior immunosuppressive therapy (45%). The primary endpoints were partial remission and complete remission. The regimen included rituximab administered over a 2-year period consisting of two initial 1000 mg IV doses separated by 2 weeks, followed by a 1000 mg IV dose every 4 months for 2 years (8 g), aimed at maintaining continuous B-cell depletion. This was combined with a short course of low-dose oral cyclophosphamide (2.5 mg/kg daily for 1 week, followed by 1.5 mg/kg daily for 7 weeks) and a rapid prednisone taper [20].

The authors reported that 100% of patients achieved partial remission, and 83% reached complete remission at 2 years. Notably, the response was early: 86% of patients achieved early immunological remission after 3 months, with 100% reaching immunological remission by 6 months. Early clinical remissions were observed in 40% of patients after 3 months, with the median time to partial remission being 3.4 months and the time to complete remission 12.4 months. Additionally, 90% of patients remained relapse-free at 2 years after onset of B-cell reconstitution following the final rituximab dose. Finally, an acceptable safety profile was described.

In comparison, the MENTOR trial (Multicenter Randomized Controlled Trial of Rituximab versus Cyclosporine in the Treatment of Idiopathic Membranous Nephropathy) using rituximab (1000 mg IV, two doses 2 weeks apart, repeated at 6 months) achieved partial remission in 60% and complete remission in 35% at 2 years [1] (see also Table 1). In the GEMRITUX trial, which used 375 mg/m^2^ rituximab doses bi-weekly, 65% and 19% of patients reached partial and complete remission, respectively [21]. Most patients in these trials showed B-cell reconstitution by 6 months, while nearly all patients in the multitarget therapy group maintained undetectable B cells throughout treatment.

**Figure 2:**
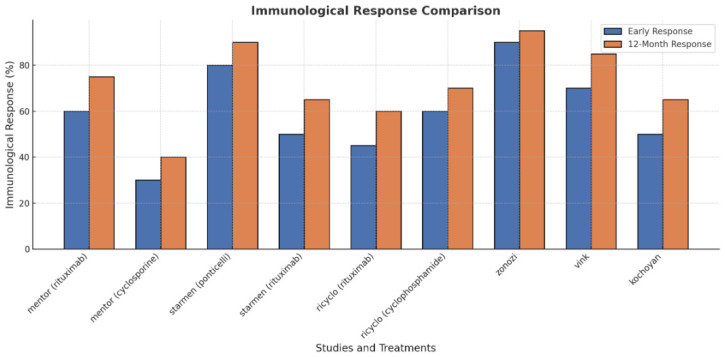
Immunological response comparison in membranous nephropathy.

In terms of immunological remission, 86% and 100% of patients achieved remission at 3 and 6 months, respectively, with similar results reported in the STARMEN (Sequential Treatment with Tacrolimus and Rituximab Versus Alternating Corticosteroids and Cyclophosphamide in primary MN) trial, where the cyclophosphamide-glucocorticoid regimen showed 77% and 92% remission rates at 3 and 6 months [22] (see also Figure 2). Rituximab monotherapy led to immunological remission in around 50% of patients at 6 months in both MENTOR and GEMRITUX. Notably, earlier declines in anti-PLA2R antibody levels, observed with cyclophosphamide-glucocorticoid therapy, predict better treatment outcomes.

**Figure 3:**
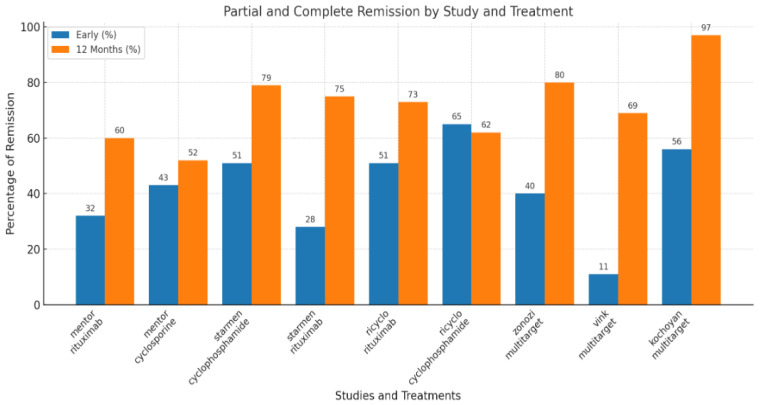
Main outcome (partial and complete response) in membranous nephropathy.

This aggressive protocol described by Zonozi et al. indeed demonstrated high rates of complete responses and relatively few relapses in membranous glomerulonephritis, but it involved significant treatment intensity, including a cumulative rituximab dose of 8 g and 26 weeks of steroid use (Figure 3). It is unclear whether the superior efficacy of triple therapy, compared to rituximab monotherapy, is due to the addition of cyclophosphamide or the higher cumulative dose of rituximab (8 g) and prolonged B-cell depletion, as opposed to the regimen with 4 g rituximab used in the MENTOR trial. Undeniably, other studies suggest that resistant patients with MN may benefit from supplementary rituximab doses or more severe B-cell depletion, such as with obinutuzumab [23].

Can similar outcomes be achieved with less intensive therapy? Vink et al. modified the low-dose regimen used in vasculitis (rituximab 2 × 1000 mg, cyclophosphamide 1.5 mg/kg/day for 8 weeks, and prednisone [2 × 1 g IV + 3 weeks oral starting at 1 mg/kg]) in a single-arm, prospective study [24]. The study included 26 patients with PLA2Rab-associated MN, a median age of 57 years, nephrotic syndrome (proteinuria 7.1 g/day, serum albumin 18 g/L), and preserved kidney function. The regimen led to rapid immunological remission in 88% of patients within 8 weeks and clinical remission of proteinuria in 73%. Anti-PLA2R antibodies decreased by more than 75% by week 2 and over 93.5% by week 4, with this rapid reduction correlating strongly with both early immunological response and clinical remission.

Recently, Kochoyan and Dobronravov reported comparable results in their study, which prospectively included patients with MN who received triple therapy at significantly lower doses. The regimen consisted of rituximab (1–2 doses of 375 mg/m^2^), cyclophosphamide (4 IV infusions of 7.5 mg/kg every other week: weeks 1, 3, 5, and 7), and prednisone (starting dose of 60 mg/week, tapering by 10 mg/week, followed by 5 mg/day from weeks 8 to 48). The study included 30 patients with PLA2Rab-associated MN, median age 45 years, nephrotic syndrome (proteinuria 11 g/day, serum albumin 20.4 g/L), and preserved kidney function (eGFR 80 mL/min/1.73 m^2^). The primary endpoint was early remission within 12 months. The authors compared the outcomes with historical controls who received either rituximab-based therapy (RTX, n = 15) or cyclosporine + corticosteroids (CSA, n = 42). Overall, partial and complete remission was observed in 17 patients (57%) at 3 months and 29 patients (97%) at 12 months. Complete remission rates were 7% at 3 months and 60% at 12 months. The median time to first remission was 2.8 months (range 1.6–3.9), and the median time to complete remission was 8.1 months (range 6–10.7). Notably, the early clinical remission rate was higher with the combined therapy compared to those treated with rituximab or cyclosporine alone. This suggests that multitarget therapy effectively combines the benefits of both approaches: a rapid immunological response with cyclophosphamide and sustained immunological and clinical response with rituximab. However, this study has specific limitations—retrospective, modest number of patients, insufficient incident patients, and in comparison, with historical controls, very few patients were treated with rituximab monotherapy and only at a low dose [25].

In an insightful editorial accompanying this study, Logt and Wetzels compared the various approaches and concluded that while Zonozi et al.’s study clearly demonstrated the efficacy of their triple therapy regimen, it is apparent that this treatment may not be suitable for all patients, as it could lead to overtreatment for some. On the other hand, the study by Kochoyan and Dobronravov suggests that similar results might be achieved with a less intensive therapy. However, further validation is necessary before drawing definitive conclusions [26].

**Table 1:** Comparative analysis in membranous nephropathy.

	MENTOR [1]	STARMEN [23]	RI-CYCLO [2]	Zonozi [21]	Vink [25]	Kochoyan and Dobronravov [26]
**Design**	Multicenter North America	Multicenter Europa	Multicenter Italy and Switzerland	Single-center retrospective	Single arm, prospective cohort study	Single-center, prospectively; comparison with historical cohorts
**N**	130	86	74	60	26	30
**Comparison**	Rituximab (2×, 1000 mg weeks 0, 2, 26, and 28 or cyclosporine (3.5 mg/kg/day for 12 months)	Modified Ponticelli vs Tacrolimus + rituximab (1 g at mo 6)	Modified Ponticelli vs rituximab (1 g) on days 1 and 15)	Rituximab 1000 mg weeks 0 and 2, months 4, 8, 12, 16, 20, and 24; cyclophosphamide 2.5 mg/kg 1 week + 1.5 mg/kg 7 weeks + prednisone	Rituximab 2 × 1000 mg, Cyclophosphamide 1.5 mg/kg/day × 8 weeks + prednisone	Rituximab 1–2 doses of 375 m/m^2^, cyclophosphamide (4 biweekly infusions of 7.5 mg/kg) and prednisone
**Inclusion criteria**	Proteinuria > 5 g/24 h eGFR > 40 mL/min	Proteinuria > 4 g/24 h eGFR > 45 mL/min	Proteinuria > 3.5 g/24 h eGFR > 30 mL/min	Median UPCR 8.4 eGFR median 64 mL/min 18 patients—eGFR 30–60 mL/min 8 patients—eGFR < 30	Median UPCR 7.1 Serum creatinine 128 µmol/L	Proteinuria-11 g/day, eGFR 80 mL/min/1.73 m^2^)
**PLA2R positivity**	74%	77%	66%	73%	100%	100%
**Early outcome**						
**Immunological remission**	50% vs. 46%	77% vs. 45%	63% vs. 50%	86%	78%	43%
**Partial response**	32% vs. 43%	51% vs. 28%	51% vs. 65%	40%	11%	56.7%
**12-month outcomes**						
**Immunological remission**	66 vs. 30%	88% vs. 79%	62% vs. 56%	100%	89%	80%
**Partial response**	60% vs. 52%	79% vs. 75%	73% vs. 62%	80%	69%	97%
**SAE**	10 vs. 17/100 pts/year	19 vs. 1	14 vs. 19%	7.9/100 pts/year	4 pts	1 pts

Immunological remission—enzyme-linked immunosorbent assay < 14 RU/mL or immunofluorescence test negative; PR—partial remission—urinary protein:creatinine ratio < 3 g/10 mmol and ≥50% decrease in proteinuria. eGFR—estimated glomerular filtration rate; PLA2R—phospholipase A2 receptor; SAE—severe adverse events.

An induction regimen involving rituximab, a short course of low-dose oral cyclophosphamide, and a rapid prednisone taper was used in 129 patients with ANCA-associated vasculitis (AAV), 70% of whom had myeloperoxidase (MPO)-ANCA and 9% had relapsing disease [27]. The median time to complete remission was 4 months, with 84% achieving remission by 5 months. Prednisone was successfully tapered by 8 months. Through the year succeeding remission, one major relapse happened. In conclusion, this combination therapy was effective, facilitated rapid tapering of glucocorticoids, and was well tolerated. The 2024 KDIGO guidelines for AAV treatment recommend a combination of rituximab, cyclophosphamide, and glucocorticoids for induction therapy, particularly in patients with markedly reduced or rapidly declining eGFR [28].

The complementary alternative pathway plays an essential role in the pathogenesis of ANCA-associated vasculitis. The development of avacopan, an oral selective C5a receptor antagonist, marks a significant progression in the treatment of AAV by targeting this pathway. When combined with rituximab or cyclophosphamide, avacopan outperformed prednisone tapering in achieving remission at week 52 and led to improvements in quality of life, eGFR recovery, and fewer glucocorticoid-related complications [29]. A post hoc analysis of the ADVOCATE trial showed that both avacopan and prednisone taper treatments resulted in similar substantial improvements in disease control across multiple domains, including general health, nervous system, mucous membranes/eyes, and skin [30].

Zonozi et al. conducted a multicenter retrospective cohort analysis of 92 patients who received avacopan for the treatment of new or relapsing AAV, alongside rituximab in 48% or a combination of rituximab and low-dose cyclophosphamide in 47% of cases. This study, the largest postmarketing evaluation of avacopan, reported high remission rates—90% at week 26 and 84% at week 52 [31]. The study included patients who were excluded from the ADVOCATE trial, such as those with advanced kidney disease, kidney replacement therapy, or those who had received plasmapheresis. Avacopan was shown to have an acceptable safety profile in these real-world applications.

The “four-hit hypothesis” of IgAN pathogenesis summaries a process that begins with the production of galactose-deficient IgA1, triggering an antiglycan autoimmune response and the formation of IgA immune complexes; these complexes accumulate in the glomeruli and stimulate mesangial cell proliferation, extracellular matrix production, cytokine release, and complement activation, leading to glomerular and tubulointerstitial inflammation and in the end, irreparable kidney damage [32]. B-cell activating factor (BAFF) and A proliferation-inducing ligand (APRIL) are key mediators in the production of galactose-deficient IgA1 and its corresponding autoantibodies, each playing essential roles in the survival and maintenance of B cells and humoral immunity. Elevated serum levels of both BAFF and APRIL are observed in patients with IgAN and correlate with disease severity [33]. Atacicept is a dual anti-B-cell activation factor proliferation-inducing ligament fusion protein. The phase IIb ORIGIN study enrolled 116 individuals with biopsy-proven IgA nephropathy and demonstrated that ataticept significantly reduced the urine protein–creatinine ratio by 36 weeks (35% reduction compared to placebo), alongside a marked decrease in circulating galactose-deficient IgA1 levels (by 60% versus placebo) [34]. In the succeeding long-term open-label extension, 113 IgAN patients were followed for an additional 60 weeks. Results showed a sustained reduction in UPCR (−52 ± 5% from baseline) and galactose-deficient IgA1 levels (−66 ± 2%). Ataticept was well tolerated, with no increased risk of infections, suggesting its potential as a safe and effective therapeutic option for IgAN [35].

Recently, it was discovered that anti-nephrin autoantibodies have a considerable role in podocytopathies, precisely in minimal change disease (MCD) and primary FSGS [36]. Their role is still controversial and, unfortunately, infeasible to measure at this moment in routine practice. Hengel et al. described these antibodies in 44% of adults with MCD, 9% of those with primary FSGS, and 52% of children with idiopathic nephrotic syndrome [37]. The prevalence of autoantibodies is augmented among patients with minimal change disease or idiopathic nephrotic syndrome who had active disease. The authors highlight the clinical utility of antinephrin autoantibody measurements in the case of a patient with minimal change disease who suffered three relapses of nephrotic syndrome. The presence/the quantitative level of antinephrin autoantibodies was closely linked with variations in the urinary albumin-to-creatinine ratio. Immunosuppressive treatment with glucocorticoids and cyclosporine A determined a transient remission, while rituximab induced complete and sustained remission, as indicated by undetectable antinephrin autoantibodies and the lack of proteinuria.

In conclusion, traditional therapies for glomerulonephritis (GN) often fail to achieve complete remission in many patients and are associated with severe side effects, such as infections, bone marrow suppression, and nephrotoxicity. Multitarget therapies, by combining agents with synergistic effects and reducing individual drug doses, may help mitigate toxicity while maintaining efficacy. Studies have shown that multitarget regimens often outperform standard treatments in terms of immunological and clinical remission rates. While promising, these therapies are not universally effective, and it is essential to identify the optimal dosage and patient population that will benefit most. Ongoing research on biomarkers will be key to personalizing treatments and balancing efficacy and safety. The era of a “one-size-fits-all” approach in GN treatment is over, and it is critical to design studies that emphasize individualized strategies. Additionally, the integration of new therapeutic modalities, such as exosome-based therapies or small molecules targeting podocytes, will likely revolutionize GN management. For now, multitarget therapies remain a promising strategy, offering hope for improved remission rates and reduced complications in patients with glomerulonephritis.

## Data Availability

The data presented in this study are available on request from the corresponding author.

## Abbreviation

GN	Glomerulonephritis
MCD	Minimal change disease
FSGS	Focal segmental glomerulosclerosis
MN	Membranous nephropathy
IgAN	Immunoglobulin A nephropathy
ANCA	Antineutrophil cytoplasmic antibody
anti-GBM	Anti-glomerular basement membrane
LN	Lupus nephritis
MMF	Mycophenolate mofetil
APOL1	*Apolipoprotein L1*
TRCP	Transient receptor potential channels
ROS	Reactive oxygen species
eGFR	Estimated glomerular filtration rate
Anti-TNF	Anti-tumor necrosis factor
BAFF	B-cell activating factor
APRIL	A proliferation-inducing ligand
IL-2	Interleukin 2
RCTs	Randomized controlled trial
anti-PLA2R	M-type phospholipase A2 receptor
KDIGO	Kidney Disease: Improving Global Outcomes
MPO	Myeloperoxidase
AAV	ANCA-associated vasculitis
